# Atrial natriuretic peptide modulates the proliferation of human gastric cancer cells via KCNQ1 expression

**DOI:** 10.3892/ol.2013.1425

**Published:** 2013-06-25

**Authors:** JIA ZHANG, ZHILONG ZHAO, CHAO ZU, HAIJIAN HU, HUI SHEN, MINGXIN ZHANG, JIANSHENG WANG

**Affiliations:** Department of Surgical Oncology, First Affiliated Hospital of Medical School, Xi’an Jiaotong University, Xi’an, Shaanxi 710061, P.R. China

**Keywords:** atrial natriuretic peptide, KCNQ1, proliferation, gastric cancer

## Abstract

Atrial natriuretic peptide (ANP) and brain NP (BNP) belong to the NP family that regulates mammalian blood volume and blood pressure. ANP signaling through NP receptor A (NPR-A)/cyclic guanosine 3′5′-monophosphate (cGMP)/ cGMP-dependent protein kinase (PKG) activates various downstream effectors involved in cell growth, apoptosis, proliferation and inflammation. Evidence has shown the critical role of plasma K^+^ channels in the regulation of tumor cell proliferation. However, the role of ANP in the proliferation of gastric cancer cells is not clear. In the present study, the expression of NPR-A in the human gastric cancer cell line, AGS, and the effect of ANP on the proliferation of AGS cells were investigated using western blotting, immunofluorescence, qPCR and patch clamp assays. The K^+^ current was also analyzed in the effect of ANP on the proliferation of AGS cells. NPR-A was expressed in the human gastric cancer AGS cell line. Lower concentrations of ANP promoted the proliferation of the AGS cells, although higher concentrations decreased their proliferation. Significant increases in the levels of cGMP activity were observed in the AGS cells treated with 10^−10^, 10^−9^ and 10^−8^ M ANP compared with the controls, but no significant differences were observed in the 10^−7^ and 10^−6^ M ANP groups. The patch clamp results showed that 10^−9^ M ANP significantly increased the tetraethylammonium (TEA)- and 293B-sensitive K^+^ current, while 10^−6^ M ANP significantly decreased the TEA- and 293B-sensitive K^+^ current. The results showed that 10^−10^ and 10^−9^ M ANP significantly upregulated the expression of potassium voltage-gated channel, KQT-like subfamily, member 1 (KCNQ1) at the protein and mRNA levels, although 10^−7^ and 10^−6^ M ANP significantly downregulated the expression of KCNQ1. The data indicated that lower and higher concentrations of ANP have opposite effects on the proliferation of AGS cells through cGMP-dependent or -independent pathways. KCNQ1 upregulation and downregulation by lower and higher concentrations of ANP, respectively, have separate effects on the promotion and inhibition of proliferation.

## Introduction

Atrial natriuretic peptide (ANP) signaling occurs through NP receptor A (NPR-A) by increasing cyclic guanosine 3′,5′-monophosphate (cGMP) and activating cGMP-dependent protein kinase (PKG). Activated PKG in turn up-regulates the expression of genes encoding ion transporters and transcription factors, which together affect cell growth, apoptosis, proliferation and inflammation (1–3). NPR-A has been reported to be expressed in lung, prostate and ovarian cancer. NPR-A expression and signaling is important for tumor growth, and a NPR-A deficiency has been shown to protect C57BL/6 mice from lung, skin and ovarian cancers, suggesting that NPR-A is a new target for cancer therapy (4). NPR-A has been demonstrated to be expressed in pre-implantation embryos and embryonic stem (ES) cells and has a novel role in the maintenance of self-renewal and the pluripotency of ES cells (5).

Evidence has demonstrated the critical role of plasma K^+^ channels in the regulation of tumor cell proliferation (6,7). It has been shown that the delayed rectifier potassium channel subunits, voltage-gated potassium channed (Kv), Kv1.3, Kv1.5, Kv1.6, Kv2.1 and Kv2.2, are expressed in human gastric cancer cells, and that the downregulation of this expression significantly inhibits the proliferation of gastric cancer (8). Gastric cancer is a common malignant disease worldwide, with a high incidence and mortality and a five-year relative post-treatment survival rate of <25%. ANP has been reported to inhibit the proliferation of various types of cancer (9). The involvement of guanylyl cyclase (GC)-coupled natriuretic receptors has been identified, with lower concentrations of ANP able to stimulate proliferation in neural tumor cell lines with the involvement of a GC receptor, while higher concentrations of ANP exert a mitogen-activated protein kinase-dependent antiproliferative action, which involves a non-GC receptor (10). Based on clinical data, it has been suggested that ANP is cardioprotective at a plasma concentration of 10^−9^ M (11). Studies have demonstrated the protective effects of exposure to 10^−9^ M ANP in endothelial cells, neural cells and hepatocytes (12–14). However, higher concentrations of ANP induce apoptosis in endothelial cells and neonatal rat cardiac myocytes (15,16). In cardiomyocytes, various effects of ANP have been shown, with the prevention or induction of apoptosis at concentrations of 10^−9^ or 10^−6^ M, respectively. The mechanism by which 10^−6^ M ANP promotes cardiomyocyte survival is the cGMP-dependent nuclear accumulation of zyxin and Akt (11).

As NPR-A is a fairly new target for cancer therapy (4), NPR-A expression in gastric cancer has not been investigated. The present study investigated the effects of the expression of NPR-A on the gastric cancer AGS cell line, the effects of ANP on the proliferation of AGS and the role of K^+^ channels in this ANP-affected proliferation.

## Materials and methods

### Chemicals and antibodies

ANP, tetraethylammonium chloride (TEA) and 293B were obtained from Sigma-Aldrich (St. Louis, MO, USA). The primary antibody was a rabbit anti-NPR-A polyclonal antibody (1:300 dilution; Abcam, Cambridge, MA, USA). The secondary antibody used was donkey anti-rabbit Alexa 488 (Molecular Probes, Eugene, OR, USA). Nuclei were stained with Hoechst 33342.

### Human gastric cancer AGS cell culture

Human gastric adenocarcinoma AGS cells (ATCC, Manassas, VA, USA) were grown in F-12k (ATCC) supplemented with 10% fetal bovine serum and 1% penicillin-streptomycin. The cells were cultured at 37°C with humidified 5% CO_2_, fed with fresh medium every third day and split when subconfluent.

### Immunofluorescence, RT-PCR and western blotting

The cells were fixed by incubation for 30 min at room temperature with 100 *μ*l of freshly prepared 3–4% paraformaldehyde in PBS (dissolved in boiling PBS and cooled to room temperature). The cells were then rinsed twice with PBS for 5 min and permeabilized by incubation at room temperature for 30 min with 3% Triton X-100 in PBS. Subsequently, the cells were rinsed three times with PBS and incubated for 15 min at room temperature with block medium (Cas-block). The cells were then incubated for 1 h at room temperature with the primary antibody in PBS containing 3% horse serum. Following incubation, the cells were rinsed three times with PBS for 5 min and further incubated with a fluorescent antibody (Anti-rabbit 488) diluted 1:500 in PBS containing 3% horse serum. The cells were then rinsed three times with PBS and incubated with 2 *μ*g/ml Hoechst 33342 for 15 min at room temperature. Next, the cells were rinsed three times in PBS and mounted onto glass slides. Subsequent to coverslipping, the cells were visualized at ×400 magnification using fluorescence microscopy.

RNA was isolated from the human gastric cancer AGS cell line according to the manufacturer’s instructions (RNeasy Plus Micro kit, Qiagen, Hilden, Germany). The purity of the RNA was estimated by the A_260_/A_280_ ratio (NanoDrop 1000 Spectrophotometer; Thermo Scientific, Waltham, MA, USA). Total RNA (200 ng) was reverse transcribed using the oligo dT primer and superscript III first strand synthesis system (Invitrogen, Carlsbad, CA, USA). PCR was subsequently performed using human potassium voltage-gated channel, KQT-like subfamily, member 1 (KCNQ1) primer pairs and platinum Taq DNA polymerase (Invitrogen). The primers were designed using Primer-Blast provided by the NCBI. The size of the PCR products was determined by comparison to a 100 bp DNA ladder (Invitrogen) under UV illumination following 1–2% agarose gel electrophoresis.

Total protein samples from the cell lysates prepared from the AGS cells were used to assess the expression of NPR-A and KCNQ1 by western blotting. In brief, 5 *μ*g of protein was fractionated by SDS-PAGE (4–20% gradient gel; Bio-Rad, Hercules, CA, USA) and transferred onto a PVDF membrane. The membrane was probed with a primary antibody against human NPR-A (Abcam, Cambridge, MA, USA), KCNQ1 HERG, kv2.1, Kv4.1 and Kv1.5 (Santa Cruz Biotechnology, Inc., Santa Cruz, CA, USA) followed by an HRP-conjugated anti-rabbit secondary antibody (Sigma-Aldrich). Protein signals were detected using the enhanced chemiluminescence (ECL) system (Thermo Scientific).

### BrdU cell proliferation assay

Cell proliferation was measured using 5-Bromo-2′-deoxy-uridine Labeling and Dectection kit III (Roche Applied Science, Mannheim, Germany) in accordance with the manufacturer’s instructions. Briefly, 1×10^4^ cells were plated into a 96-well plate and grown to confluence. Prior to incubation with ANP, the cells were serum free for 24 h. Subsequently, the cells were incubated with various concentrations of ANP for 24 h. The medium was switched to culture medium containing 10 *μ*M BrdU and the cells were incubated for an additional 2 h. BrdU incorporation into cellular DNA was measured using a microplate reader (Safire II; Tecan, Männedorf, Switzerland). Three independent experiments were performed and each assay was performed in triplicate.

### cGMP assay

cGMP levels were measured as previously described (17). Briefly, 1×10^5^ cells were plated in each 35-mm dish and grown to confluence. The cells were washed with 1 ml medium, then incubated for an additional 15 min at 37°C with or without ANP at concentrations of 10^−10^, 10^−9^, 10^−8^, 10^−7^ or 10^−6^ M. The medium was then replaced by 0.3 ml 0.45% NP-40 (Sigma-Aldrich). Subsequent to a 5-min incubation on ice, the lysate was removed from the plates and centrifuged for 2 min at 4°C (12,000 × g). The supernatant was collected and assayed for cGMP levels using a cGMP ELISA kit (Cell Biolabs, San Diego, CA, USA) according to the manufacturer’s instructions.

### Patch clamp recordings

The voltage clamp technique was performed using whole-cell configuration at room temperature (22–25°C). The Tyrode’s solution used in the experiments contained the following: 140 mM NaCl, 5.4 mM KCl, 1.2 mM MgCl_2_, 5 mM HEPES, 1.8 mMx CaCl_2_ and 10 mM glucose, which was titrated to pH 7.4 with NaOH. The pipette solution used in the experiments contained the following: 120 mM K-Asparatate, 10 mM Na_2_ATP/2H_2_O, 2 mM MgCl_2_, 10 mM EGTA and 10 mM HEPES, which was titrated to pH 7.4 with KOH. The glass pipette electrodes were made from Corning 7056 glass capillaries (Warner Instruments, Hammed, CT, USA) with a pipette resistance of 2–3 MΩ in the bath solution. All recordings were initiated at least 10 min after the rupture of the membrane. Signals were measured with an Axopatch 700A amplifier using pCLAMP 9 software (Molecular Devices, Sunnyvale, CA, USA), with a Bessel low-pass filter (cut-off frequency, 10 kHz) and a sampling frequency of 10 kHz.

### Statistical analysis

All data were analyzed using Clampfit (Axon Instruments, Sunnyvale, CA, USA) and Igor software (WaveMetrics, Lake Oswego, OR, USA). P<0.05 was considered to indicate a statistically significant difference.

## Results

### NPR-A is expressed in the human gastric cancer AGS cell line

**Figure 1.**
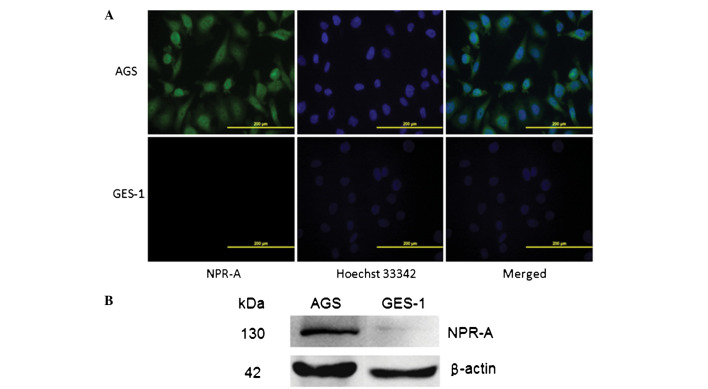
(A) NPR-A expression in human gastric cancer AGS cells. AGS cells were stained with NPR-A antibody and visualized at ×400 magnification. Nuclei were stained with Hoechst 33342. (B) NPR-A protein expression in AGS gastric cancer cells and human gastric epithelial immortalized GES-1 cells was determined by western blot analysis. Anti-β-actin was used as a loading control. Final images are cropped to highlight relevant bands. NPR-A, natriuretic peptide receptor A. *P<0.05.

NPR-A expression was evaluated by western blotting and immunofluorescence in the human gastric cancer AGS cells and was compared with human gastric epithelial immortalized GES-1 cells. The results of the western blot analysis and immunofluorescence showed that NPR-A was expressed abundantly in the human gastric cancer AGS cells, but not in the human gastric epithelial immortalized GES-1 cells (Fig. 1).

### Effect of ANP on the proliferation of the AGS cells

**Figure 2.**
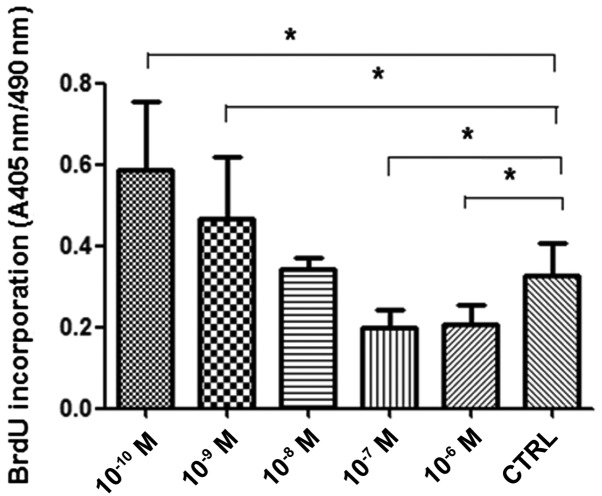
AGS cells treated with various concentrations of ANP for 24 h. Cell proliferation was performed using 5-Bromo-2′-deoxy-uridine Labeling and Detection kit III according to the manufacturer’s instructions.^*^P<0.05.

The effect of ANP on AGS cell proliferation was evaluated by comparing the BrdU incorporation into replicating DNA in the human gastric cancer AGS cells. The results showed that 10^−10^ and 10^−9^ M ANP significantly promoted the proliferation of the AGS cells (Fig. 2, P<0.05, n=3), while 10^−7^ and 10^−6^ M ANP significantly inhibited the proliferation of the AGS cells (Fig. 2; P<0.05; n=3). No significant differences were detected between the 10^−8^ M ANP and control groups (Fig. 2; P>0.05; n=3). The results in the AGS cells of the present study were similar to those Kato *et al* observed in cardiomyocytes (11). Lower concentrations of ANP promote the proliferation of AGS cells, while higher concentrations decrease the proliferation of AGS cells.

### cGMP assay

**Figure 3.**
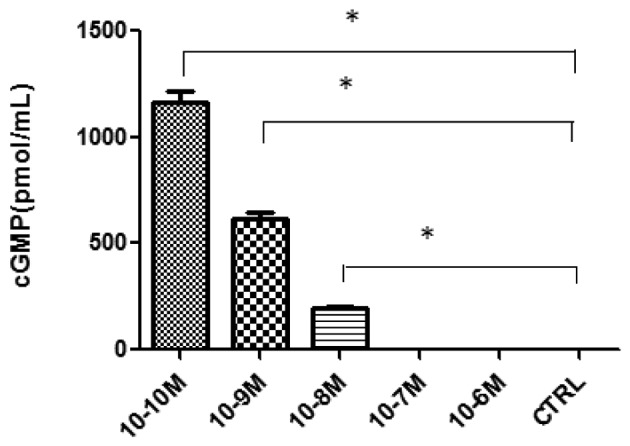
Intracellular levels of cGMP measured by cGMP analysis. AGS cells were treated with 10^−10^, 10^−9^,10^−8^, 10^−7^ and 10^−6^ M ANP for 15 min, then cell proteins were collected for cGMP assay. ^*^P<0.05. Data were obtained from three independent experiments. cGMP, cyclic guanosine 3′,5′-mono-phosphate.

A cGMP ELISA kit assay was used to investigate ANP-induced changes in the cGMP levels. Fig. 3 shows that the incubation of the AGS cells with 10^−10^, 10^−9^ and 10^−8^ M ANP significantly increased the level of cGMP activity compared with the control (P<0.05; n=3), while no significant differences were observed in the 10^−7^ and 10^−6^ M ANP groups (P>0.05; n=3).

### Kv in AGS cells

**Figure 4.**
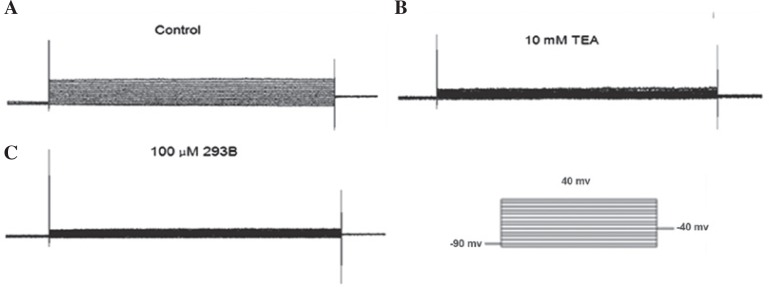
Effect of TEA and 293B on the voltage-dependent outward K^+^ current. (A) Superimposed global outward K^+^ current elicited by a depolarizing step pulse with 500 msec duration from a holding potential of −90 to 40 mV. (B) Applying 10mM TEA to the bath solution eliminated this voltage-dependent outward K^+^ current. (C) Applying 100 *μ*M 293B to the bath solution also eliminated this voltage-dependent outward K^+^ current. The data represent the mean ± SEM obtained from nine cells. TEA, tetraethylammonium chloride.

In agreement with previous studies (8), the AGS cells exhibited a prominent voltage-gated outward K^+^ current, while the membrane potential was depolarized from a holding potential of −90 to 40 mV (Fig. 4). This current was blocked completely by 10 mM TEA and 100 *μ*M 293B (Fig. 4). These results suggest that the K^+^ current (I_K_) in the AGS cells was via a TEA- and 293B-sensitive I_K_.

### I_K_ channels of AGS cells revealed by immunofluorescence and western blot analysis

**Figure 5.**
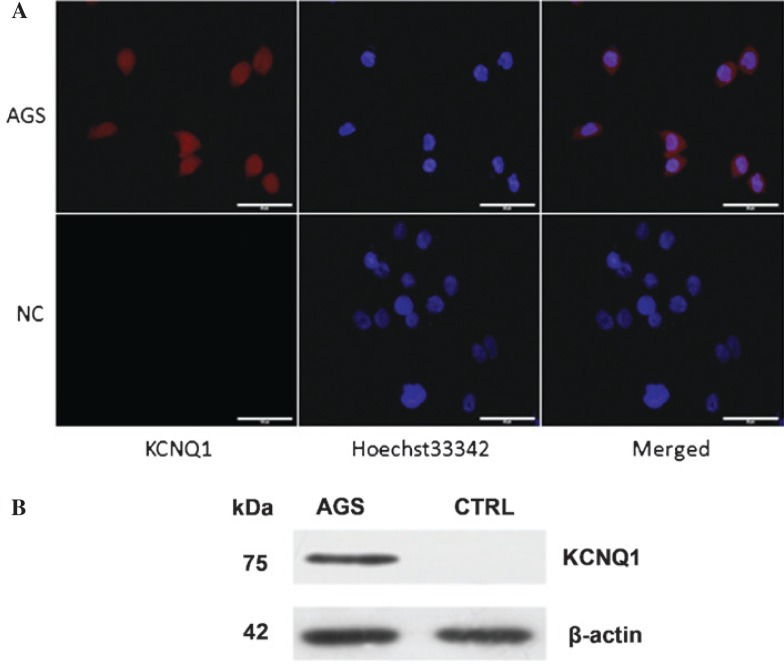
(A) KCNQ1 expression in human gastric cancer AGS cell line. AGS cells were stained with KCNQ1 antibody and visualized at ×400 magnification. Nuclei were stained with Hoechst 33342. (B) KCNQ1 protein expression in human AGS gastric cancer cells was determined by western blot analysis. Anti-β-actin was used as a loading control. Final images are cropped to highlight relevant bands. KCNQ1, potassium voltage-gated channel, KQT-like subfamily, member 1.

It has been reported that KCNQ1 (18), HERG (19–22), Kv1.3 (8), Kv1.5 (8,23), Kv1.6 (8), Kv2.1 (8), Kv2.2 (8), KCNE2 (24,25), Eag1 (26) and KATP (27) are the main I_K_ channels of AGS cells. The patch clamp results showed that the K^+^ current in the AGS cells was a 293B-sensitive I_K_. 293B is the inhibitor of the KCNQ1 channel (28). The expression of KCNQ1 in the AGS cells was investigated by immunofluorescence and western blotting. The results of the western blot analysis and immunofluorescence showed that KCNQ1 is expressed abundantly in human gastric cancer AGS cells (Fig. 5).

### ANP modulates the voltage-gated outward K^+^ current in AGS cells

**Figure 6.**
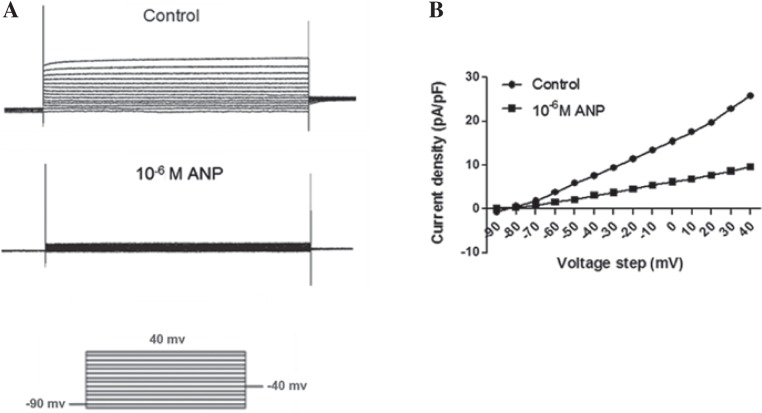
The steady-state activation property of I_K_ was decreased by 10^−6^ M ANP in the AGS cells. (A) I_K_ recordings using an activation voltage protocol in the absence (top) and presence (bottom) of 10^−6^ M ANP. The cells were held at −90 mV and depolarized in 10 mV steps with a 500 msec duration from −90 to 40 mV at 10 sec intervals. (B) Voltage-dependent activation curve of I_K_ obtained in the absence or presence of 10^−6^ M ANP. Current density (in pA/pF) is plotted against the voltage step. The data represent the mean ± SEM obtained from twelve cells. ANP, atrial natriuretic peptide.

**Figure 7.**
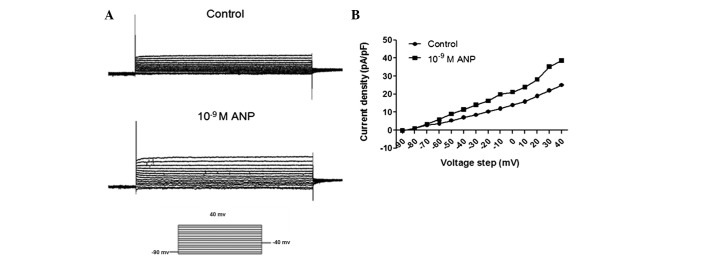
The steady-state activation property of I_K_ was increased by 10^−9^ M ANP in the AGS cells. (A) I_K_ recordings using an activation voltage protocol in the absence (top) and presence (bottom) of 10^−9^ M ANP. The cells were held at −90 mV and depolarized in 10 mV steps with a 500 msec duration from −90 to 40 mV at 10 sec intervals. (B) Voltage-dependent activation curve of I_K_ obtained in the absence or presence of 10^−9^ M ANP. Current density (in pA/pF) is plotted against the voltage step. The data represent the mean ± SEM obtained from twelve cells. ANP, atrial natriuretic peptide.

Using the BrdU cell proliferation assay, lower concentrations of ANP (10^−10^ and 10^−9^ M) were observed to promote the proliferation of the AGS cells, while higher concentrations of ANP (10^−7^ and 10^−6^ M) decreased proliferation. According to the present data and previous studies, 10^−9^ M ANP was selected as the lower concentration, while 10^−6^ M ANP was used as the higher concentration for the patch clamp study. The effect of ANP on voltage-dependent steady-state activation and inactivation was then studied at concentrations of 10^−9^ and 10^−6^ M ANP. The steady-state activation of I_K_ was elicited using the appropriate voltage protocols as follows: I_K_ was evoked by a 500 msec depolarizing pulse from a first pulse potential of −90 to 40 mV, in 10 mV steps at 10 sec intervals (Figs. 6A and 7A). The TEA- and 293B-sensitive K^+^ current was significantly increased by 10^−9^ M ANP (n=12, P<0.05), while 10^−6^ M ANP significantly decreased the TEA- and 293B-sensitive K^+^ current (n=12, P<0.05). By plotting normalized conductance as a function of the command potential, the I_K_ activation curve was obtained. As shown in Figs. 6B and 7B, the activation curve was significantly shifted towards the left by the application of 10^−9^ M ANP. The activation curve was significantly shifted towards the right by the application of 10^−6^ M ANP (Fig. 6B).

**Figure 8.**
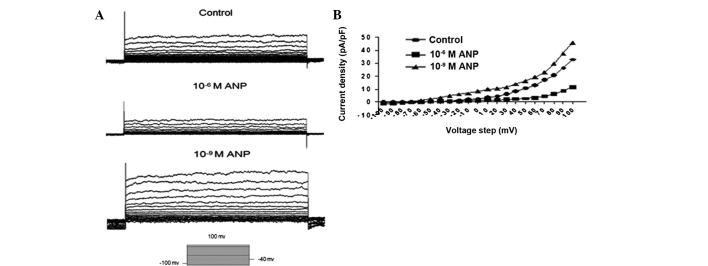
ANP modified the steady-state activation property of I_K_ in the AGS cells. (A) I_K_ recordings from human gastric cancer AGS cells using an activation voltage protocol in the control and 10^−6^ M ANP and 10^−9^ M ANP groups for 24 h. The cells were held at −100 mV and depolarized in 10 mV steps with a 500 msec duration from −100 to 100 mV at 10 sec intervals. (B) Voltage-dependent activation curve of I_K_ obtained in the absence or presence of ANP. Current density (in pA/pF) is plotted against the voltage step. The data represent the mean ± SEM obtained from twenty cells. ANP, atrial natriuretic peptide.

In the BrdU cell proliferation assay, the AGS cells were incubated at various concentrations of ANP for 24 h. In the patch clamp study, the AGS cells were only treated for 3–5 min to investigate the effects of various concentrations of ANP on the voltage-gated outward K^+^ current. Next the AGS cells were incubated with various concentrations of ANP for 24 h. Subsequent to this, the AGS cells were used for a patch clamp study. I_K_ was evoked by a 500 msec depolarizing pulse from a first pulse potential of −90 to 40 mV, in 10 mV steps at 10 sec intervals (Fig. 8A). The results were similar to when the AGS cells were treated for 3–5 min to investigate the effects of various concentrations of ANP on the voltage-gated outward K^+^ current. The TEA- and 293B-sensitive K^+^ current was significantly increased by 10^−9^ M ANP (n=12, P<0.05), while 10^−6^ M ANP significantly decreased the TEA- and 293B-sensitive K^+^ current (n=12, P<0.05). By plotting the normalized conductance as a function of the command potential, the I_K_ activation curve was obtained. As shown in Figs. 7B and 8B, the activation curve was significantly shifted towards the left by the application of 10^−9^ M ANP, while the activation curve was significantly shifted towards the right by the application of 10^−6^ M ANP.

### Effect of ANP on the expression of KCNQ1

**Figure 9.**
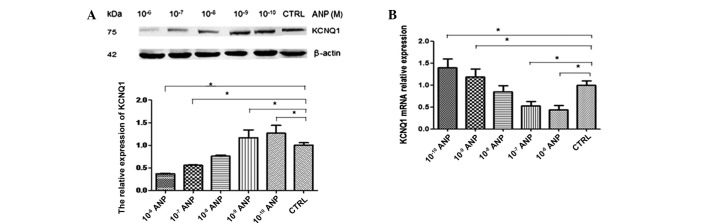
AGS cells treated with various concentrations of ANP for 24 h. (A) KCNQ1 protein expression levels detected by western blotting. Data are expressed as the mean ± SEM of each group of cells from three separate experiments, and the values of the control cells were designated. ^*^P<0.05. (B) AGS cells treated with various concentrations of ANP for 24 h. KCNQ1 mRNA expression levels were detected by qPCR. Data are expressed as the mean ± SEM of each group of cells from three separate experiments. ^*^P<0.05. ANP, atrial natriuretic peptide; KCNQ1, potassium voltage-gated channel, KQT-like subfamily, member 1.

The AGS cells were treated with various concentrations of ANP for 24 h. KCNQ1 protein expression levels were detected by western blotting. Data are expressed as the mean ± SEM of each group of cells from three separate experiments. As shown in Fig. 9A, 10^−10^ and 10^−9^ M ANP significantly upregulated the expression of KCNQ1 at the protein level (n=3; P<0.05), while 10^−7^ and 10^−6^ M ANP significantly downregulated expression (n=3; P<0.05). KCNQ1 mRNA expression levels were detected by qPCR. The qPCR results were similar to those obtained by western blotting. As shown in Fig. 9B, 10^−10^ and 10^−9^ M ANP significantly upregulated the expression of KCNQ1 at the mRNA level (n=3; P<0.05), while 10^−7^ and 10^−6^ M ANP significantly downregulated expression (n=3; P<0.05).

## Discussion

Using immunofluorescence, BrdU assays and whole-cell patch clamp recording, it was revealed that NPR-A is expressed in the human gastric cancer AGS cell line and that lower and higher concentrations of ANP have opposing effects on the proliferation of AGS cells. The voltage-gated outward K^+^ current was demonstrated to be involved in the anti-proliferative effect of higher concentrations of ANP and the pro-proliferative effect of lower concentrations of ANP.

NPR-A is the receptor for ANP and brain NP (BNP). ANP and BNP belong to the NP family, which regulates mammalian blood volume and blood pressure. ANP signaling through NPR-A/cGMP/PKG activates various downstream effectors involved in cell growth, apoptosis, proliferation and inflammation (2). NPR-A has been reported to be expressed in lung, prostate and ovarian cancer. NPR-A expression and signaling is important for tumor growth and its deficiency has be shown to protect C57BL/6 mice from lung, skin and ovarian cancers, suggesting that NPR-A is a new target for cancer therapy (4,29). In the present study, using the immunofluorescence method, it was demonstrated that NPR-A is expressed in AGS cells.

In neural tumor cell lines, the involvement of guanylyl cyclase (GC)-coupled natriuretic receptors has been identified, with lower concentrations of ANP able to stimulate proliferation with the involvement of a GC receptor, while higher concentrations of ANP exert a mitogen-activated protein kinase-dependent antiproliferative action, which involves a non-GC receptor (10). Another study demonstrated similar results in cardiomyocytes (11). In the present study, as NPR-A is expressed in AGS cells, the effect of ANP was investigated on the proliferation of the AGS cells. The results obtained were similar to those from the neural tumor cell lines and cardiomyocyte studies (10,11).

Since plasma K^+^ channels are critical in the regulation of tumor cell proliferation, typical high (10^−6^ M) and low (10^−9^ M) concentrations of ANP were used to investigate its effect on the K^+^ channels of AGS cells. The results showed that 10^−6^ M ANP significantly decreased the TEA- and 293B-sensitive K+ current, while 10^−9^ M ANP significantly increased the TEA- and 293B-sensitive K^+^ current.

According to a review of the literature and the present study results, this K^+^ current is 293B-sensitive. 293B is the inhibitor of the KCNQ1 channel. Consequently, the decision was made to focus on KCNQ1. The expression of KCNQ1 was investigated in the AGS cells by immunofluorescence and western blotting. The results of the western blot analysis and immunofluorescence showed that KCNQ1 is expressed abundantly in human gastric cancer AGS cells. According to the present data and results from previous studies, 10^−9^ M ANP was selected as the lower concentration, while 10^−6^ M ANP was selected as the higher concentration for the patch clamp study. The patch clamp results showed that 10^−9^ M ANP significantly increased the TEA- and 293B-sensitive K+ current, while 10^−6^ M ANP significantly decreased the TEA- and 293B-sensitive K^+^ current. To investigate the role of KCNQ1 in the effects of various concentrations of ANP on the proliferation of AGS cells, the AGS cells were treated with various concentrations of ANP for 24 h. KCNQ1 protein and mRNA expression levels were detected by western blotting and qPCR, respectively. The results showed that, at the protein and mRNA level, 10^−10^ and 10^−9^ M ANP significantly upregulated the expression of KCNQ1, while 10^−7^ and 10^−6^ M ANP significantly downregulated expression.

The present data indicated that lower and higher concentrations of ANP have opposing effects on the proliferation of AGS cells through cGMP-dependent or -independent pathways. KCNQ1 upregulation and downregulation by lower and higher concentrations of ANP, respectively, have separate effects on the promotion and inhibition of proliferation.
